# Hybrid Theory^†^: Creation of a Faculty Position That Allows Half-Time Hospice Employment Alongside Academic Palliative Care

**DOI:** 10.1089/pmr.2022.0013

**Published:** 2022-10-04

**Authors:** Michael Cafarchio, Dierdre Torto Salanger, W. Anthony Riley, Amanda VanderZyl, Catherine Y. Hamel, Thomas J. Smith, Danielle J. Doberman

**Affiliations:** ^1^ Section of Palliative Medicine, Division of General Internal Medicine, Department of Medicine, Johns Hopkins School of Medicine, Baltimore, Maryland, USA.; ^2^ Henry Ford Hospital, Detroit, Michigan, USA.; ^3^ Gilchrist Services, Towson, Maryland, USA.; ^4^ Department of Medicine, Johns Hopkins University, Baltimore, Maryland, USA.; ^5^ Department of Oncology, Sidney Kimmel Comprehensive Cancer Center, Johns Hopkins University, Baltimore, Maryland, USA.

**Keywords:** academic advancement, hospice, palliative care, strategic alliance

## Abstract

**Background:**

The specialty of hospice and palliative medicine struggles to merge the fast-paced technological consultative work of acute hospital palliative care with slower paced and home-based hospice. Each has equal if different merits. Here, we describe creation of a position that allowed half-time hospice employment, alongside academic hospital-based palliative care.

**Methods:**

Johns Hopkins Medicine and Gilchrist, Inc., a large nonprofit hospice, partnered to form a joint position with time spent equally between the two locations.

**Results:**

Created as a university position with “leasing” to the hospice, specific attention has been paid to mentoring at both sites to allow professional advancement. Both organizations have benefited in terms of recruitment, and more physicians have chosen this dual pathway suggesting that it is working well.

**Discussion:**

Hybrid positions are possible and may be desired by those who wish to practice both palliative medicine and hospice. Creation of one successful position helped recruit a second and a third candidate a year later. The original recipient has been promoted within Gilchrist to direct the inpatient unit. Such positions require careful mentoring and coordination to allow success at both sites and this can be done with foresight.

## Introduction

Hospice and palliative medicine (HPM) became an American Board of Internal Medicine recognized specialty in 2006 and is continuing to grow and mature. To sit for the certification examination, graduates must complete a 12-month clinical fellowship including rotations in home and inpatient hospice, ambulatory palliative care, and hospital-based consultative practice. Fellowship programs struggle to expose trainees to the diverse patient population and systems of serious illness care, while also achieving the depth of knowledge and experience needed to guide ultimate career choice. In the context of an increasing workforce shortage of HPM practitioners,^1^ it is essential to equip new providers for lifelong competency in the various aspects of our field.

The possible tension between the worlds of fast-paced technologically driven academic palliative medicine and community-based hospice practice presents a challenge for new job seekers. At the end of fellowship, one traditionally chooses between these solitudes. What happens if an HPM professional enjoys both HPM, and wants to maintain an academic position? Under the traditional service-based hospital model, working in a community hospice setting may not contribute significantly to promotion or other academic advancement. In addition, the variety in practice patterns produces both benefits and challenges for a new attending physician.

When working as a consultant in the hospital environment, the locus of control is often less direct than in other practice settings. Thus, a consultative practice requires cultivation of advanced communication skills to collaborate with peer physicians and partner with multidisciplinary stakeholders to create a unified plan of care. In contrast, serving as the attending of hospice record in the home or inpatient setting allows physicians the most control over the medical plan of care, including even complex critical care cases such as ventilator withdrawal. This may satisfy a physician's intellectual curiosity and desire for locus of control; however, the ability to teach learners, have an academic peer group, and benefit from grand rounds and research supports are not always robust in this environment.

We undertook this study to record the pathway to creation of an academic hybrid position for one of our graduating fellows. To our knowledge, this is the first such reported successful effort and we report it in the hope that other programs may find it instructive.

## Methods

### Defining the participants

Today, Gilchrist Hospice (GH) is Maryland's largest hospice provider serving >800 patients daily. Originally founded in 1994 as a nonprofit home care hospice called the Hospice of Baltimore, GH adopted its current name in 2008, honoring founding benefactor, Jeanne “Jinny” Gilchrist Vance. The organization's first inpatient unit, a 24-bed facility now known as Gilchrist Center Towson, opened in 1996. As a result of a higher demand for inpatient hospice care, in 2009, it expanded from 24 to 34 beds and later opened a second inpatient hospice center in Columbia, Maryland—Gilchrist Center Howard County.

More recently, in 2014, Gilchrist began managing the only residential inpatient hospice center in Baltimore, now named Gilchrist Center Baltimore. In 2011, Gilchrist was awarded the American Hospital Association Circle of Life award. By providing financial support to two HPM fellows at Johns Hopkins each year, Gilchrist helps build the HPM workforce and recruits highly qualified physicians.

The palliative care program at Johns Hopkins was started in the mid-2000s with a small program in the Johns Hopkins Comprehensive Cancer Center and in the Medical Intensive Care Unit (ICU). The program has now grown to six advanced practice nurses, three nurses embedded in various high-need programs, five full-time and seven part-time physicians, two part-time pharmacists, and two social workers. Over 1000 unique new patients are served annually between the inpatient and outpatient settings, with total visits nearing 10,000. The fellowship started in 2012 and typically has four or five fellows, including pediatric HPM fellows.

### Where did the idea originate?

Dr. Doberman stated the issue: “As a Fellowship Program Director, mentoring fellows through the job hunt when they are applying to a position both with your program and externally is very much like being a parent; you simultaneously want them to stay and to spread their wings to explore other opportunities. In this case, we had a fellow interested in taking a position with us as an inpatient consult attending, while also considering a position as an inpatient hospice attending, amongst other offers.

With only several weeks exposure to the different job environments, graduating fellows look to program leadership to provide unbiased counsel on the pros and cons of these opportunities. I was also keenly aware of the need for well-trained staff at both Gilchrist and Johns Hopkins, especially in the role of academic site director at the inpatient hospice center, which we depend on to help train fellows and rotating residents. I chose to call the medical director of the hospice (GH) and explore the general notion of a shared position under the concept: ‘better that we share him, than we both lose him to a job elsewhere.’”

The palliative program was approved for incremental physician hires to support the mission of Johns Hopkins. GH was also recruiting for physicians in the face of ongoing personnel shortages. Physician recruitment is handled in partnership with Johns Hopkins physician leadership, an administrative representative, and the candidate. Through this process, it became evident that the candidate's ideal position would include clinical work in both the hospice and palliative spaces to continue building on both aspects of fellowship training and glean the benefits of academic medicine. As GH is a formal rotation for the HPM fellowship, the fellow and hospice physicians had mutual familiarity before start of negotiations.

The idea was proposed to Johns Hopkins (program administrator) by Dr. Anthony Riley to explore a shared position between Johns Hopkins and GH after preliminary conversations with the candidate. Johns Hopkins explored the possibility from a contracting perspective with the Johns Hopkins Clinical Practice Association's legal counsel. The most feasible arrangement was determined to be one in which Johns Hopkins hired the candidate, with the external entity (GH) financially supporting the agreed upon effort back to Johns Hopkins.

## Results

### The contract

After broad-stroke negotiations in which both parties agreed that the idea was sound, the arrangement was deemed to be mutually beneficial for Johns Hopkins and GH. For the academic center, initially one and now three inpatient palliative care consultation experts were acquired. The hospice center gained inpatient practitioners experienced in critical care medicine and hospice, ideally suited to serve an increasingly complex patient population, often transferring directly from local ICUs, a growing national trend.^2^ Importantly, the new hires took on an academic leadership role at the hospice unit by being the primary preceptors for fellows and residents on their hospice rotations, and the first participant has now become the medical director at GH inpatient unit.

The contract was initiated by the academic center—in essence, a full year contract like any other, with “leasing” of the physician to the hospice. The usual requirements for academic advancement in the “clinician-educator” track still apply; to date, all physicians have been in that track rather than in the traditional academic track. It is too early in their career to know about promotion.

## Discussion

Entrepreneurship in health care is often undervalued. In the original review of nine early palliative care programs, a common theme was an individual or group of people who decided “We are just going to *do* this.”^3^ We believe this represents an example of a strategic alliance,^4^ a transparent agreement that benefits both parties. The hospice gains experienced medical help with a regular schedule, as well as a recruiting tool for other health care professionals. The academic center gains experienced medical help with a regular schedule, as well as the opportunity to showcase hospice as a critical component of the HPM fellowship program.

Both sides benefit from the enhanced ability to teach medical trainees about HPM career opportunities. Ultimately, the patient and family benefit from having physicians highly skilled in the hospital-to-hospice transition, both as individual providers and as a knowledgeable resource to colleagues.

As with all new initiatives, there are lessons learned. The candidate must be willing to start a new model and meet the employment requirements of both the hospice and the academic center, including credentialing at multiple institutions. The partners in the strategic alliance must maintain open communication about scheduling and meeting the contract requirements. Learned lessons in logistics include how to divide vacation time and to monitor two e-mails. The hybrid provider must also remain unbiased when making referrals from the hospital to area hospices. In this case, the hospice and the academic center have a longstanding favorable relationship.

Negotiations about the contract likely pose more difficulty for the academic center. HPM physicians are in high demand, so salaries must be adjusted upward from those in general academic internal medicine (while maintaining some semblance of equity). Though there is not yet enough longitudinal experience, we are hopeful these physicians will advance along the academic faculty track with adequate mentoring.

We believe this may serve as a model for other institutions and persons seeking to build careers in both hospice- and hospital-based palliative care.

## Funding Information

All TJS - Supported by grants NCI grant P 30 006973; 1 R01 CA177562-01A1, PCORI IHS 1609-36518; the Harry J. Duffey Family Fund for Palliative Care; The Lerner Foundation, Washington, DC.

## Abbreviations Used

HPM: hospice and palliative medicine
GH: Gilchrist Hospice
ICU: intensive care unit
